# Electrophysiological Indices of Response Inhibition in a Go/NoGo Task Predict Self-Control in a Social Context

**DOI:** 10.1371/journal.pone.0079462

**Published:** 2013-11-12

**Authors:** Kyle Nash, Bastian Schiller, Lorena R. R. Gianotti, Thomas Baumgartner, Daria Knoch

**Affiliations:** Social and Affective Neuroscience, Department of Psychology, University of Basel, Switzerland; University of Bern, Switzerland

## Abstract

Recent research demonstrates that response inhibition—a core executive function—may subserve self-regulation and self-control. However, it is unclear whether response inhibition also predicts self-control in the multifaceted, high-level phenomena of social decision-making. Here we examined whether electrophysiological indices of response inhibition would predict self-control in a social context. Electroencephalography was recorded as participants completed a widely used Go/NoGo task (the cued Continuous Performance Test). Participants then interacted with a partner in an economic exchange game that requires self-control. Results demonstrated that greater NoGo-Anteriorization and larger NoGo-P300 peak amplitudes—two established electrophysiological indices of response inhibition—both predicted more self-control in this social game. These findings support continued integration of executive function and self-regulation and help extend prior research into social decision-making processes.

## Introduction

Executive functions are cognitive mechanisms that direct the dynamics of thought and action [1]. Core functions include a working memory component that holds and updates relevant information, a set shifting component that allows switching between tasks or information sets, and a response inhibition component that acts as the brake on dominant, automatic, or prepotent behavior [2]. Intriguingly, research has begun to reveal that these fundamental executive functions may subserve broad self-regulation and self-control processes [3]. For example, working memory capacity predicts regulation of anger and resistance to tempting stimuli [4] and response inhibition predicts better control of smoking behavior [5]. Essentially, these putatively ‘cool’ executive functions appear to interface with ‘hot’ motivational processes. A key question remains. Do basic executive functions like response inhibition also predict self-regulation and self-control in social decision-making?

In comparison to reigning in a motor-response or resisting a tempting snack, however, self-control in social decision-making is much more complex. The goals or impulses are abstract (e.g., goals that comply with social norms or goals that promote personal achievement) and social decisions often require consideration of another’s mental state (i.e., theory of mind, [6]). It is thus questionable that response inhibition also predicts self-control in such a multifaceted, high-level phenomenon as social decision-making. Preliminary research suggests that it may. In one study, performance on a stop-signal task predicted strategic responding in an ultimatum game [7]. However, reaction time tasks are not direct measures of ongoing response inhibition processes (i.e., there is no response to index). As such, we utilized electroencephalography (EEG) to examine whether neural activity associated with response inhibition would predict complex decision-making in a social game that would require self-control.

We employed an economic exchange game with real, monetary consequences (referred to as a broken promise game). In this paradigm, participants promised whether or not they would return money to ostensibly real partners, but were later given the opportunity to break that promise. Critical to understanding whether a response requires self-control is whether or not a prepotent impulse must be inhibited at the decision point [8]. We designed the game in such a way that the prepotent response was to follow through with the promise to return money. Thus, the response that required self-control was breaking the promise. Importantly, this notion has empirical support. In a functional magnetic resonance imaging study, breaking a promise was associated with increased activation in the dorsolateral prefrontal cortex (PFC) and the anterior cingulate cortex (ACC), among other regions, suggesting that participants recruited these control-related regions to inhibit the promised response to return money [9].

To index response inhibition processes, we measured two established electrophysiological indices based on the P300 event-related potential (ERP) during a Go/NoGo task called the cued Continuous Performance Test (CPT). The NoGo-P300 potential is known to peak over fronto-central electrodes at approximately 300 to 500 ms after stimulus presentation [10]. From this ERP, we calculated both the NoGo Anteriorization (NGA, [11]) and peak amplitude of the NoGo-P300. The NGA is a comparison of EEG topographical maps between NoGo- and Go-ERPs at the P300 peak after stimulus presentation (e.g., [11]). The NoGo-ERPs consistently show a forward-shift or ‘anteriorization’ of the positive centroid (i.e., the ‘center of gravity’ of the positive electrical field on the scalp). Higher NGA values are thought to reflect increased frontal activation recruited to control or inhibit the prepotent motor-response. Indeed, the NGA is reduced in patients characterized by a ‘disinhibited’ pathology, including attention deficit/hyperactivity disorder, schizophrenia, genetic risk alleles, and 22q11.2 deletion syndrome [12]–[16]. Additionally, increased NGA has been associated with increased baseline activation in lateral PFC regions associated with response inhibition and cognitive control [17]. The NoGo-P300 peak amplitude, like the NGA, is thought to specifically reflect response inhibition [18], [19]. For example, the NoGo-P300 is elicited by both motor and cognitive inhibition and its amplitude is sensitive to increased inhibitory load or demand [19], [20]. NoGo-P300 peak amplitude has also been related to pathologies characterized by impulsivity [21].

We expected that response inhibition processes would predict self-control in a social context. In the broken promise paradigm, the prepotent response to be inhibited at the decision point was following through with the promise to return an investment. Breaking the promise thus required self-control. Consequently, we hypothesized that a larger NGA and larger NoGo-P300 peak amplitudes would both predict the degree to which participants broke their promises. Further, we examined whether source-localized neural activity specific to response inhibition would also be associated with the degree to which participants broke their promises.

## Methods

### Ethics Statement

This study was approved by the Ethikkommission Beider Basel (EKBB) of the University of Basel. All subjects gave written informed consent before the study.

### Participants and Procedure

In the first of two sessions, 45 right-handed participants (age *M* = 23.58, *SD* = 5.01; 26 females, years of education *M* = 16.86, *SD* = 2.83) each completed the CPT during which EEG was recorded. All subjects were screened for health problems with a detailed questionnaire. They had no current or prior history of neurological or psychiatric disorder and no history of alcohol or drug abuse. Note that one person was excluded from analyses based on outlier NGA data and regression influence statistics (*Cook’s Distance*  = 0.67, over 8 times larger than the next highest value), leaving 44 participants for analyses. Participants completed the broken promise game in a second session in small groups on separate computers. Subjects received 40 Swiss francs (CHF 40; CHF 1 ∼ $1 U.S.) compensation for participating, in addition to money earned in the broken promise game.

### Response Inhibition Task: Cued Continuous Performance Test (CPT)

To elicit response inhibition, we utilized the CPT [22], [23]. In this task, participants prepare and implement a speeded button press to particular ‘target’ stimuli and inhibit the prepared response to ‘non-target’ stimuli. Letters were presented centrally on a computer screen one letter at a time for 200 ms (inter-stimulus interval: 1650 ms) in a pseudo-randomized order. Before the task, participants were instructed to press the response button on ‘Go’-trials—a paired sequence of stimuli in which the letter O (a primer stimuli) was first presented then followed by the letter X (a target stimulus). On ‘NoGo’-trials, participants were instructed to not respond when the letter O was followed by any letter other than X (a non-target stimulus). Participants were finally instructed to give their answers as quickly and as accurately as possible. The stimulus set consisted of 400 trials (with 12 different letters: A, B, C, D, E, F, G, H, J, L, O, X), of which 80 were primer stimuli, 40 were target stimuli, and 40 were non-target stimuli. The remaining stimuli were 240 distractor letters (letters other than O, or an X without a preceding O). Because target and non-target stimuli are equally probable, the comparison of brain responses between NoGo- and Go-stimuli is not confounded by oddball or frequency of stimuli effects [24], [25], allowing us to directly examine the electrophysiology of inhibiting versus executing a motor response.

### Electrophysiological Measurement

Continuous EEG was recorded from 64 Ag-AgCl active electrodes positioned according to the 10/10 system montage [26]. EEG was sampled at 512 Hz (24 bit precision; bandwidth: 0.1–100 Hz) and was referenced to common mode sense with a driven right leg ground. Horizontal and vertical electro-oculographic signals were recorded with electrodes at the left and right outer canthi and left infraorbital muscle. Eye-movement artifacts were corrected by independent component analysis. EEG signals from channels with corrupted signals were interpolated.

### Event-Related Potentials Processing: NGA and P300

EEG data from the CPT were first filtered offline with a bandpass from 0.1 to 30 Hz. An automatic artifact detection within an epoch of 200 ms before to 1000 ms after stimulus presentation marked amplitudes greater than 70 µV. Data were then visually inspected to detect any residual artifacts. All available artifact-free EEG epochs from correct responses were segmented, re-referenced (to an average reference of all electrodes), baseline corrected (using a -200 ms - 0 ms pre-stimulus window as baseline), and individually averaged to Go- and NoGo-ERPs (number of artifact-free Go-epochs: *M* = 34.30, *SD* = 4.40; number of artifact-free NoGo-epochs: *M* = 33.70, *SD* = 5.11). All subjects had a minimum of 20 artifact-free and correct-response Go- and NoGo-epochs.

P300 peak latencies were defined at the electrode with the most positive deflection; Pz for Go-trials (240–484 ms) and Cz for NoGo-trials (304–444 ms). Time windows were derived from the P300 microstates (quantifiable time periods of relatively stable electric field configurations, for further explanation of the methodology see [27]). Go- and NoGo-P300 peak amplitudes were then indexed at the respective peak latency from all electrodes for each subject.

**Figure 1:**
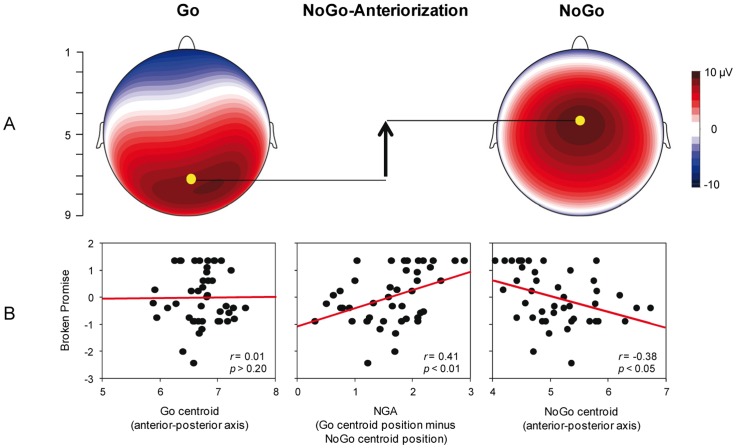
Relationship between the Broken Promise score and the NGA. A. Scalp field maps showing the positive centroid position on an anterior posterior axis (from 1 =  most anterior, to 9 =  most posterior) in the Go- (left panel) and NoGo-ERPs (right panel). The NGA was calculated as the Go-positive centroid position minus NoGo-positive centroid position (middle panel). B. Scatterplots of the correlations between the standardized Broken Promise score (i.e., larger numbers indicate a higher ratio of broken promises) and the Go-positive centroid (left), the NGA (middle), and the NoGo-positive centroid (right).

To calculate the NGA, positive area centroids of P300 field maps [28] were calculated at each individual’s P300 peak for both Go- and NoGo-ERPs. The location of each individual Go- and NoGo-positive centroid was determined by fitting or projecting the electrode array from the scalp onto a rectangular coordinate system. Positive centroid locations were measured on an anterior-posterior scale ranging from 1 (position of the electrode Fpz) to 9 (position of Oz; see Figure 1). Smaller values thus indicate a more anterior centroid. The NGA was calculated for each subject as the difference between Go- and NoGo-positive centroids on this anterior-posterior axis [11] such that more positive numbers indicate a larger NGA.

### Source Localization of Inhibition-Related Neural Activity

We utilized standardized low-resolution brain electromagnetic tomography (sLORETA; [29]) to estimate intracerebral activation during response inhibition in the CPT. sLORETA computes electric neuronal activity as current density (A/m^2^) without assuming a predefined number of active sources. The sLORETA solution space consists of 6,239 voxels (voxel size: 5×5×5 mm) and is restricted to cortical gray matter and hippocampi, as defined by the digitized Montreal Neurological Institute (MNI) probability atlas. Using the option automatic regularization method in the sLORETA software, we chose the transformation matrix with the signal-to-noise set to 10.

Specifically, we wished to identify brain regions that significantly contributed to the NGA and NoGo-P300. Thus, sLORETA images were computed at individual P300 peaks for Go- and NoGo-conditions, respectively. To reduce confounds that have no regional specificity, for each subject and for each condition, sLORETA images were normalized to a total power of one and then log-transformed before statistical analyses.

### Broken Promise Game

We adapted a basic trust game in which two players, interacting anonymously, played the roles of an investor (Player A) and a trustee (Player B). Our subjects were always in the role of Player B. Player B first makes a ‘promise’ at the beginning of three subsequent game trials, indicating whether he or she will ‘never’, ‘sometimes’, ‘mostly’ or ‘always’ (given the values from 0-3, respectively) return Player A’s investment. At the start of a single trial, Player A is informed about Player B’s promise level and decides whether to invest money (2 money units, MUs) or not. If Player A does invest, MUs are increased fivefold (10 MUs). Player B then decides to either send back half (5 MUs) or keep the full amount (MUs were exchanged at the end of the study for real money, 1 MU  =  CHF 1/5). Thus, Player B can either keep his or her promise or break it. The experiment consisted of 9 trials with three promise decisions from Player B preceding three subsequent trials. Player B thus played in 9 separate trials with 9 different, anonymous, and randomly selected interaction partners. We report the average promise level of the three promise decisions and the average investment return rate across all return decisions.

A Broken Promise score was then calculated as the ratio of investment return level (number of returns/number of investments from Player A) to promise level (sum of promise values/9 [i.e., total possible sum of promise values]). This variable was then reflected and standardized for easier interpretation and plotting, such that a higher number indicates that the person returned proportionately less in comparison to promise levels, whereas a lower number indicates that the person returned proportionately more in comparison to promise levels.

### Statistical Analyses

To examine whether self-control in social decision-making is related to response inhibition we entered the Broken Promise score into separate Pearson correlations with both the NGA and the NoGo-P300 peak amplitude. We also correlated both the Go- and NoGo-positive centroid positions with the Broken Promise score to further corroborate that it is a shift towards frontal regions in the NoGo-ERP that is associated with self-control in this task. Additionally, because the NoGo-P300 peak amplitude is maximal at fronto-central electrodes, we restricted our analyses to the following central-midline positions for NoGo-P300 peak amplitudes: CPz, Cz (the maximal peak in these data), and FCz.

Lastly, we examined whether source-localized brain activity related to response inhibition was associated with the Broken Promise score. We contrasted the sLORETA images of the NoGo- versus Go-condition at individual P300 peaks and regressed this contrast on the Broken Promise score. As it has been consistently shown that frontocingulate regions encompassing the lateral PFC and ACC are more active during the NoGo- compared with the Go-condition [30]–[32], we restricted this voxel-by-voxel regression analysis to all voxels encompassing prefrontal regions (Brodmann areas [BAs] 8, 9, 10, 11, 44, 45, 46, and 47; 1331 voxels) and anterior cingulate regions (BAs 24, 32, and 33; 313 voxels). Correction for multiple testing (for all voxels of the frontocingulate regions) was implemented by means of a nonparametric randomization approach [33]. The nonparametric randomization approach was used to estimate empirical probability distributions and the corresponding corrected (for multiple comparisons) critical probability thresholds.

## Results

In the CPT, participants made an average of 0.89 (*SD* = 0.95) errors, whereas the modal error total was 0. More specifically, participants made, on average, 0.48 (*SD* = 0.82) omission errors (no response to Go-stimuli) and 0.41 (*SD* = 0.58) commission errors (incorrect response to any stimuli other than Go-stimuli). Average reaction time to Go-stimuli was 389.86 ms (*SD* = 84.79). Consistent with prior research on the NGA [22], topographical analysis revealed that the positive centroid was more anterior in the NoGo-ERP (coordinate position *M* = 5.10, *SD* = 0.65) compared to the Go-ERP (coordinate position *M* = 6.72, *SD* = 0.50; *t*(43)  = 17.62, *p* <0.001, see Figure 1).

Examination of behavior in the broken promise game revealed that even though participants reported very high promise levels (*M* = 2.61, *SD* = 0.50; 54% chose ‘3 - always’ to return the investment for each promise round, and 93% chose at least ‘2 - almost always’ for each round [i.e., chose a value of 2 or higher]), there was considerable variability in actual investment return levels (return rate: *M* = 51%, *SD* = 35%). The most frequent return rate was to return nothing in all rounds (21%), whereas the next most frequent return rate was to always return the investment (18%). Thus, there was a high degree of variance in deceptive behavior (similar to prior results, [9]). We also examined whether decision times differed between breaking a promise and keeping a promise. As in prior research on decision times, we focused on the first decision to minimize in-game learning and interaction history effects (e.g., [34]). A one-way ANOVA revealed that people who broke their promise on the first decision displayed longer decision times (*M* = 6.08 s, *SD* = 3.41) than people who kept their promise (*M* = 4.22 s, *SD* = 0.92), *F*(1, 39)  = 7.58, *p*<0.01. Thus, breaking a promise required the most time, indicative of increased deliberation and self-control [35].

**Figure 2:**
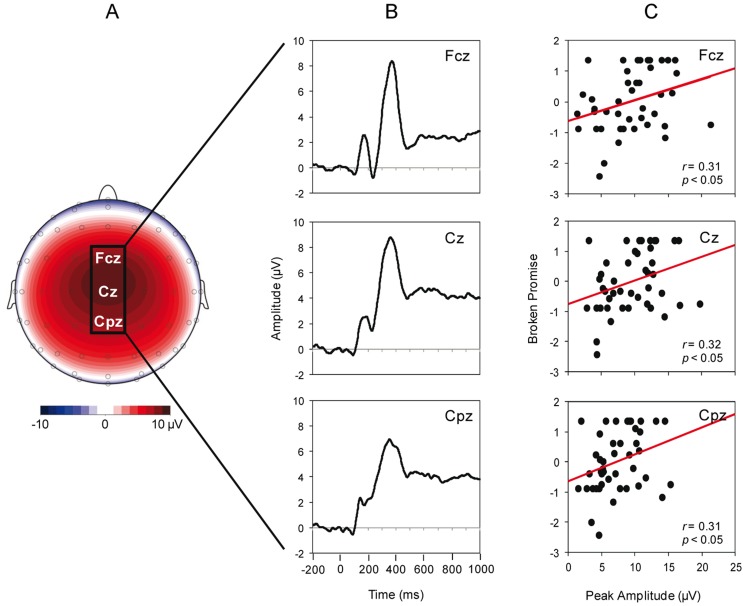
Relationship between the Broken Promise score and the NoGo-P300 amplitudes. A. Approximate scalp position of the electrodes used for the NoGo-P300 peak amplitude, FCz, Cz, CPz. B. Plot of the NoGo-ERPs (stimulus presentation at 0 ms). C. Scatterplots of the correlation between the Broken Promise score (i.e., larger numbers indicate a higher ratio of broken promises) and the NoGo-P300 peak amplitudes.

In our primary analyses, we examined correlations of the NGA and the NoGo-P300 peak amplitude with the Broken Promise score. In support of our prediction that neural markers of the executive function response inhibition would predict self-control in social decision-making, the NGA was significantly related to the Broken Promise score. That is, greater NGA values were associated with a higher Broken Promise score, *r*(42)  = 0.41, *p*<0.01. Moreover, this correlation appears attributable primarily to the NoGo-positive centroid position (*r*(42)  = –0.38, *p*<0.05) and not the Go-positive centroid position (*r*(42)  = 0.01), indicating that the Broken Promise score was correlated with a more anterior NoGo-positive centroid as opposed to a more posterior Go-positive centroid (note that the correlation is negative here because the coordinate system ranges from 1 – most anterior to 9 – most posterior, see Figure 1). This directly refutes the notion that the NGA-Broken Promise correlation is due to processes in the Go-condition. Moreover, much like the NGA finding, NoGo-P300 peak amplitude was also correlated with the Broken Promise score; at FCz, *r*(42)  = 0.31, *p*<0.05; at Cz, *r*(42)  = 0.32, *p*<0.05; at CPz, *r*(42)  = 0.31, *p*<0.05 (see Figure 2). Thus, two different electrophysiological indices of response inhibition were associated with the Broken Promise score.

**Figure 3:**
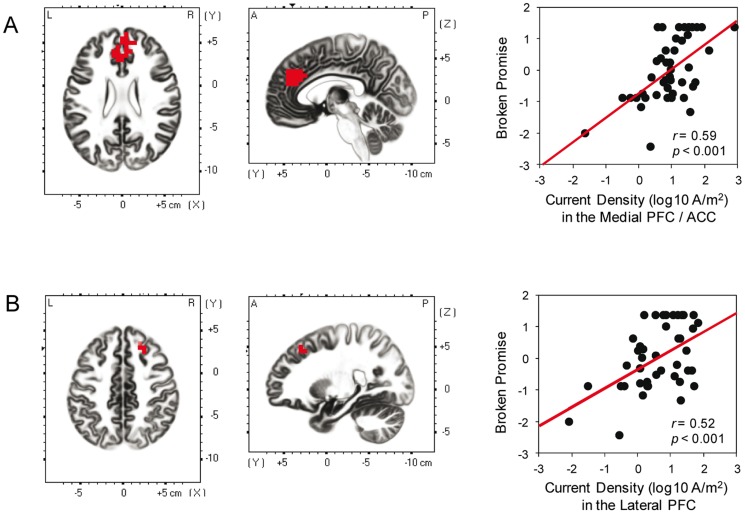
Relationship between the Broken Promise score and source-localized brain activity related to response inhibition. In the first two panels on the left, locations of the voxels that showed significant correlations are indicated in red (*p*<0.05, corrected) and, on the right, scatterplots are shown demonstrating the relationship between the Broken Promise score and source-localized brain activity (i.e., demonstrating the average correlation across all voxels that exceeded the corrected *p* threshold in the same cluster). We found significant positive correlations between the Broken Promise score and current density in the medial PFC/ACC (A; BAs 9, 10, and 32, peak voxel at MNI [x, y, z] –5, 40, 25), and in the lateral PFC (B; BA 8, peak voxel at MNI [x, y, z] 25, 30, 45).

Finally, source-localization analysis showed that higher Broken Promise scores were associated with greater activation during response inhibition in the medial PFC/ACC, *cluster average r*(42) =  0.59, *p*<0.001 (BAs 9, 10, and 32, peak voxel: MNI [x, y, z] −5, 40, 25, see Figure 3A), and the right lateral PFC, *cluster average r*(42)  = 0.52, *p*<0.001 (BA 8, peak voxel: MNI [x, y, z] 25, 30, 45, see Figure 3B). Consistent with the NGA and NoGo-P300 scalp results, the source-localized brain activity specifically related to response inhibition was associated with the Broken Promise score. That is, neural markers of motor-response control predicted self-control in a complex social context.

## Discussion

Recent research has demonstrated that executive functions are involved in self-regulation and self-control [3]. However, a direct link between executive functions and self-control in social decision-making has rarely been demonstrated [36]. Here we examined whether electrophysiological indices of response inhibition would predict controlled behavior in a social decision-making task. We found that the NGA, NoGo-P300 peak amplitude, and response inhibition-related brain activity all predicted self-control in an economic exchange game. Moreover, the activation that was associated with the Broken Promise score was source-localized to medial PFC/ACC and lateral PFC, brain regions that are both thought to be involved in self-control across a variety of domains [37]–[40]. This study thus provides some of the first evidence that directly connects non-social with social forms of self-control.

One might suggest that the Broken Promise score reflects the reverse effect—a lack of self-control. That is, the prepotent response would be selfish, monetary gain and the score reflects a failure to inhibit this greedy impulse. We think this interpretation is unlikely for several reasons. First, past research shows that breaking a promise (specifically in this paradigm), as compared to keeping a promise, activates self-control related brain areas, namely medial PFC/ACC and lateral PFC regions [9]. Second, we assessed two established yet separable electrophysiological indices of response inhibition—the NGA and the NoGo-P300 peak amplitude—as well response inhibition-related brain activity. We supplement these findings by analyzing reaction time to Go-trials. Past research has inferred better response inhibition processes from faster reaction times to Go-stimuli [41], [42]. In the current research, much like the NGA and the NoGo-P300 peak amplitude, faster Go-trial reaction times were related to higher Broken Promise scores, *r*(42)  = –0.27, *p*<0.08. Thus, four separate indices linked to response inhibition—three electrophysiological and one behavioral—were associated with the Broken Promise score in the same direction. If the Broken promise score did reflect a lack of self-control, one would expect the opposite effects, not the effects we found. Third, we demonstrated that breaking a promise required the most time, indicative of increased deliberation and self-control. These points all converge to support our theoretical assertion that a higher Broken Promise score reflects more self-control.

These results have implications for prior evidence that self-control involves a core process. For example, self-control in one domain can impact self-control in subsequent, unrelated domains [5], [43]. Self-control is relatively stable from childhood to adulthood across a variety of situations [44]. A number of psychopathological disorders are attributed to disrupted impulse control [45]–[47]. The inhibition of motor responses, emotions, desires, and cognitions reliably involves similar brain regions (for a recent review, see [40]). Because response inhibition measures also predict self-control across various domains, including delaying gratification, thought inhibition, emotion suppression [3], [44], and now social decision-making, perhaps the core process of self-control is the executive function of response inhibition? Further, these findings are consistent with studies that examine response inhibition processes and psychopathological disorders characterized by impulsivity [45]. As these disorders often co-occur with social difficulties [47], our results corroborate the notion that the disruption of executive functions may produce social deficits [46].

More broadly, meaningful communication between the cognitive and social psychological literatures on self-regulation has been lacking until relatively recently [3]. The current research supplements the notion that this burgeoning, integrative perspective on self-regulation can unite and strengthen these separate paradigms. Drawing on both cognitive and social psychological literatures hints at intriguing new research avenues. For example, a considerable amount of social psychological research indicates that self-control can be temporarily reduced and such reductions may be due to impairment of executive functioning [36], [48]. Based on the current results, prospective research could explore whether impaired or disrupted executive functioning predicts impaired self-control in other, high-level social contexts. Alternatively, training manipulations that boost executive functioning over the long-term [49], [50] could potentially promote lasting improvements in the regulation of social behavior.

## References

[1] Baddeley AD (2007) Working memory, thought, and action. Oxford, United Kingdom: Oxford University Press.

[2] Miyake A, Friedman NP, Emerson MJ, Witzki AH, Howerter A (2000) The unity and diversity of executive functions and their contributions to complex ‘‘frontal lobe’’ tasks: A latent variable analysis. Cogn Psychol 41: 49–100. 10945922 10.1006/cogp.1999.0734

[3] Hofmann W, Schmeichel BJ, Baddeley AD (2012) Executive functions and self-regulation. Trends Cogn Sci 3: 174–180. 10.1016/j.tics.2012.01.006 22336729

[4] Hofmann W, Gschwendner T, Wiers R, Friese M, Schmitt M (2008) Working memory capacity and self-regulation: Towards an individual differences perspective on behavior determination by automatic versus controlled processes. J Pers Soc Psychol 95: 962–977. 18808271 10.1037/a0012705

[5] Berkman ET, Falk EB, Lieberman MD (2011) In the trenches of real-world self-control: Neural correlates of breaking the link between craving and smoking. Psychol Sci 22: 498–506. 21378368 10.1177/0956797611400918 PMC3076513

[6] Rilling JK, Sanfey AG (2011) The neuroscience of social decision-making. Annu Rev Psychol 62: 23–48. 20822437 10.1146/annurev.psych.121208.131647

[7] Steinbeis N, Bernhardt BC, Singer T (2012) Impulse control and underlying functions of the left DLPFC mediate age-related and age-independent individual differences in strategic social behavior. Neuron 73: 1040–1051. 22405212 10.1016/j.neuron.2011.12.027

[8] Thaler RH, Shefrin HM (1981) An economic theory of self-control. J Polit Econ 89: 392–406.

[9] Baumgartner T, Fischbacher U, Feierabend A, Lutz K, Fehr E (2009) The neural circuitry of a broken promise. Neuron 64: 756–70. 20005830 10.1016/j.neuron.2009.11.017

[10] Polich J (2007) Updating P300: An integrative theory of P3a and P3b. Clin Neurophysiol 118: 2128–2148. 17573239 10.1016/j.clinph.2007.04.019 PMC2715154

[11] Fallgatter AJ, Strik WK (1999) The NoGo-anteriorization as a neurophysiological standard-index for cognitive response control. Int J Psychophysiol 32: 233–238. 10437634 10.1016/s0167-8760(99)00018-5

[12] Dresler T, Ehlis AC, Heinzel S, Renner TJ, Reif A, et al (2010) Dopamine transporter (SLC6A3) genotype impacts neurophysiological correlates of cognitive response control in an adult sample of patients with ADHD. Neuropsychopharmacology 35: 2193–2202. 20631685 10.1038/npp.2010.91 PMC3055310

[13] Fallgatter AJ, Muller TJ (2001) Electrophysiological signs of reduced prefrontal response control in schizophrenic patients. Psychiatry Res 107: 19–28. 11472861 10.1016/s0925-4927(01)00092-0

[14] Fallgatter AJ, Ehlis AC, Rosler M, Strik WK, Blocher D, et al (2005) Diminished prefrontal brain function in adults with psychopathology in childhood related to attention deficit hyperactivity disorder. Psychiatry Res 138: 157–169. 15766638 10.1016/j.pscychresns.2004.12.002

[15] Fallgatter AJ, Herrmann MJ. Hohoff C, Ehlis AC, Jarczok TA, et al (2006) DTNBP1 (dysbindin) gene variants modulate prefrontal brain function in healthy individuals. Neuropsychopharmacology 31: 2002–2010. 16407900 10.1038/sj.npp.1301003

[16] Romanos M, Ehlis AC, Baehne CG, Jacob C, Renner TJ, et al (2010) Reduced NoGo-anteriorisation during continuous performance test in deletion syndrome 22q11.2. J Psychiatr Res 44: 768–774. 20188379 10.1016/j.jpsychires.2010.02.001

[17] Schiller B, Gianotti LRR, Nash K, Knoch D (2013) Individual differences in inhibitory control—Relationship between baseline activation in lateral PFC and an electrophysiological index of response inhibition. Cerebr Cortex. Advance online publication. doi: 10.1093/cercor/bht095 10.1093/cercor/bht095 23588188

[18] Pfefferbaum A, Ford JM, Weller BJ, Kopell BS (1985) ERPs to response production and inhibition. Electroencephalogr Clin Neurophysiol 60: 423–434. 2580694 10.1016/0013-4694(85)91017-x

[19] Smith JL, Johnstone SJ, Barry RJ (2008) Movement-related potentials in the Go/NoGo task: The P3 reflects both cognitive and motor inhibition. Clin Neurophysiol 119: 704–714. 18164657 10.1016/j.clinph.2007.11.042

[20] Enriquez-Geppert S, Konrad C, Pantev C, Huster RJ (2010) Conflict and inhibition differentially affect the N200/P300 complex in a combined go/nogo and stop-signal task. Neuroimage 51: 877–887. 20188191 10.1016/j.neuroimage.2010.02.043

[21] Szuromi B, Czobor P, Komlósi S, Bitter I (2011) P300 deficits in adults with attention deficit hyperactivity disorder: A meta-analysis. Psychol Med 41: 1529–1538. 20961477 10.1017/S0033291710001996

[22] Fallgatter AJ, Brandeis D, Strik WK (1997) A robust assessment of the NoGo-anteriorisation of P300 microstates in a cued continuous performance test. Brain Topogr 9: 295–302. 9217988 10.1007/BF01464484

[23] Rosvold HE, Mirsky AF, Sarason I, Bransome Jr ED, Beck LH (1956) A continuous performance test of brain damage. J Consult Clin Psychol 20: 343–350. 10.1037/h0043220 13367264

[24] Liddle PF, Kiehl KA, Smith AM (2001) Event-related fMRI study of response inhibition. Hum Brain Mapp 12: 100–109. 11169874 10.1002/1097-0193(200102)12:2<100::AID-HBM1007>3.0.CO;2-6 PMC6871906

[25] Lavric A, Pizzagalli DA, Forstmeier S (2004) When 'go' and 'nogo' are equally frequent: ERP components and cortical tomography. Eur J Neurosci 20: 2483–2488. 15525290 10.1111/j.1460-9568.2004.03683.x

[26] Nuwer MR, Comi G, Emerson R, Fuglsang-Frederiksen A, Guerit JM, et al (1998) IFCN standards for digital recording of clinical EEG. International Federation of Clinical Neurophysiology. Electroencephalogr Clin Neurophysiol 106: 259–261. 9743285 10.1016/s0013-4694(97)00106-5

[27] Michel CM, Koenig T, Brandeis D (2009) Electrical neuroimaging in the time domain. In: Michel CM, Koenig T, Brandeis D, Gianotti LRR, Wackermann J, editors. Electrical Neuroimaging. Cambridge: University Press, Cambridge; p. 111–143

[28] Koenig T, Gianotti LRR (2009) Scalp field maps and their characterization. In: Michel CM, Koenig T, Brandeis D, Gianotti LRR, Wackermann J, editors. Electrical Neuroimaging. Cambridge: University Press, Cambridge; p. 25–47

[29] Pascual-Marqui RD (2002) Standardized low-resolution brain electromagnetic tomography (sLORETA): Technical details. Methods Find Exp Clin Pharmacol 24(Suppl. D): 5–12. 12575463

[30] Fallgatter AJ, Bartsch AJ, Herrmann MJ (2002) Electrophysiological measurements of anterior cingulate function. J Neural Transm 109: 977–988. 12111483 10.1007/s007020200080

[31] Garavan H, Ross TJ, Murphy K, Roche RAP, Stein EA (2002) Dissociable executive functions in the dynamic control of behavior: Inhibition, error detection, and correction. Neuroimage 17: 1820–1829. 12498755 10.1006/nimg.2002.1326

[32] Swick D, Ashley V, Turken U (2011) Are the neural correlates of stopping and not going identical? Quantitative meta-analysis of two response inhibition tasks. Neuroimage 56: 1655–1665. 21376819 10.1016/j.neuroimage.2011.02.070

[33] Nichols TE, Holmes AP (2002) Nonparametric permutation tests for functional neuroimaging: A primer with examples. Hum Brain Mapp 15: 1–25. 11747097 10.1002/hbm.1058 PMC6871862

[34] Rand DG, Greene JD, Nowak MA (2012) Spontaneous giving and calculated greed. Nature 489: 427–430. 22996558 10.1038/nature11467

[35] Metcalfe J, Mischel W (1999) A hot/cool system analysis of delay of gratification: Dynamics of willpower. Psychol Rev 106: 3–19. 10197361 10.1037/0033-295x.106.1.3

[36] Heatherton TF (2011) Neuroscience of self and self-regulation. Annu Rev Psychol 62: 363–390. 21126181 10.1146/annurev.psych.121208.131616 PMC3056504

[37] Kuhn S, Haggard P, Brass M (2009) Intentional inhibition: How the “veto-area” exerts control. Hum Brain Mapp 30: 2834–2843. 19072994 10.1002/hbm.20711 PMC6870995

[38] Knoch D, Fehr E (2007) Resisting the power of temptations. Ann NY Acad Sci 1104: 123–134. 17344543 10.1196/annals.1390.004

[39] Aron AR, Robbins TW, Poldrack RA (2004) Inhibition and the right inferior frontal cortex. Trends Cogn Sci 8: 170–177. 15050513 10.1016/j.tics.2004.02.010

[40] Cohen JR, Lieberman MD (2010) The common neural basis of exerting self-control in multiple domains. In: Trope Y, Hassin R, Ochsner KN, editors. Self-Control in Society, Mind, and Brain. Oxford University Press: Oxford, UK. p. 141–160.

[41] Fallgatter AJ, Herrmann MJ (2001) Electrophysiological assessment of impulsive behavior in healthy subjects. Neuropsychologia 39: 328–333. 11163610 10.1016/s0028-3932(00)00115-9

[42] Heinzel S, Dresler T, Baehne CG, Heine M, Boreatti-Hummer A, et al.. (2012) COMT × DRD4 epistasis impacts prefrontal cortex function underlying response control. Cereb Cortex doi:10.1093/cercor/bhs132. 10.1093/cercor/bhs132 22617852

[43] Muraven M, Baumeister RF (2000) Self-regulation and depletion of limited resources: Does self-control resemble a muscle? Psychol Bull 126: 247–259. 10748642 10.1037/0033-2909.126.2.247

[44] Casey BJ, Somerville LH, Gotlib IH, Ayduk O, Franklin NT, et al (2011) Behavioral and neural correlates of delay of gratification 40 years later. Proc Natl Acad Sci USA 108: 14998–15003. 21876169 10.1073/pnas.1108561108 PMC3169162

[45] Aron AR, Poldrack RA (2005) The cognitive neuroscience of response inhibition: Relevance for genetic research in attention-deficit/hyperactivity disorder. Biol Psychiatry 57: 1285–1292. 15950000 10.1016/j.biopsych.2004.10.026

[46] Heatherton TF, Wagner DD (2011) Cognitive neuroscience of self-regulation failure. Trends Cogn Sci 15: 132–139. 21273114 10.1016/j.tics.2010.12.005 PMC3062191

[47] Robbins TW, Gillan CM, Smith DG, de Wit S, Ersche KD (2012) Neurocognitive endophenotypes of impulsivity and compulsivity: Towards dimensional psychiatry. Trends Cogn Sci 16: 81–91. 22155014 10.1016/j.tics.2011.11.009

[48] Schmeichel BJ (2007) Attention control, memory updating, and emotion regulation temporarily reduce the capacity for executive control. J Exp Psychol Gen 136: 241–255. 17500649 10.1037/0096-3445.136.2.241

[49] Houben K, Jansen A (2011) Training inhibitory control: Recipe for resisting sweet temptations. Appetite 56: 345–349. 21185896 10.1016/j.appet.2010.12.017

[50] Klingberg T (2010) Training and plasticity of working memory. Trends Cogn Sci 14: 317–324. 20630350 10.1016/j.tics.2010.05.002

